# Selection of Single-Stranded DNA Molecular Recognition Elements against Exotoxin A Using a Novel Decoy-SELEX Method and Sensitive Detection of Exotoxin A in Human Serum

**DOI:** 10.1155/2015/417641

**Published:** 2015-11-09

**Authors:** Ka Lok Hong, Kailey Yancey, Luisa Battistella, Ryan M. Williams, Katherine M. Hickey, Chris D. Bostick, Peter M. Gannett, Letha J. Sooter

**Affiliations:** ^1^Department of Pharmaceutical Sciences, West Virginia University, 1 Medical Center Drive, P.O. Box 9530, Morgantown, WV 20506, USA; ^2^School of Medicine, WV University, Morgantown, WV 20506, USA; ^3^Department of Medical Laboratory Science, West Virginia University, Morgantown, WV 20506, USA; ^4^Molecular Pharmacology & Chemistry Program, Memorial Sloan Kettering Cancer Center, 1275 York Avenue, New York, NY 10065, USA; ^5^College of Pharmacy, Nova Southeastern University, Ft. Lauderdale, FL 33328, USA

## Abstract

Exotoxin A is one of the virulence factors of *Pseudomonas aeruginosa*, a bacterium that can cause infections resulting in adverse health outcomes and increased burden to health care systems. Current methods of diagnosing *P. aeruginosa* infections are time consuming and can require significant preparation of patient samples. This study utilized a novel variation of the Systematic Evolution of Ligand by Exponential Enrichment, Decoy-SELEX, to identify an Exotoxin A specific single-stranded DNA (ssDNA) molecular recognition element (MRE). Its emphasis is on increasing stringency in directing binding toward free target of interest and at the same time decreasing binding toward negative targets. A ssDNA MRE with specificity and affinity was identified after fourteen rounds of Decoy-SELEX. Utilizing surface plasmon resonance measurements, the determined equilibrium dissociation constant (*K*_*d*_) of the MRE is between 4.2 *µ*M and 4.5 *µ*M, and is highly selective for Exotoxin A over negative targets. A ssDNA MRE modified sandwich enzyme-linked immunosorbent assay (ELISA) has been developed and achieved sensitive detection of Exotoxin A at nanomolar concentrations in human serum. This study has demonstrated the proof-of-principle of using a ssDNA MRE as a clinical diagnostic tool.

## 1. Introduction

Exotoxin A is a virulence factor secreted by Gram negative bacilli bacteria,* Pseudomonas aeruginosa *[1].* P. aeruginosa* has been identified as an opportunistic bacterium that is commonly associated with wound infections, nosocomial lung infections, and respiratory diseases in cystic fibrosis patients [2, 3]. Due to increasing antibiotic resistance, infections caused by* P. aeruginosa* have been associated with decrease in the quality of life, increased mortality in patients, and significant cost burden in health care systems [4, 5].

Upon covalent cleavage of the full length protein, the enzymatically active fragment of Exotoxin A enters host cells [6]. It causes ADP-ribosylation of elongation factor 2 and thus inhibits polypeptide assembly to ribosome and protein translation, causing death of host cells [7, 8]. Early studies of purified Exotoxin A report an intravenous lethal dose as low as 3 *μ*g/kg in mice or a LD_50_ of ~10 *μ*g/kg via intraperitoneal injection [9, 10]. Because of this highly toxic nature, it is essential to treat* P. aeruginosa* infection as early as possible.

However, current diagnosis of* P. aeruginosa* infection largely relies on traditional methods, such as Gram-stain, bacterial culturing, biochemical methods, and immunoassays [11]. Though those methods are sensitive and reliable, they require a significant amount of time to confirm infection, prolonging the time between patient clinical presentations and antibiotics treatments. This leads to the initial use of nonspecific broad spectrum antibiotics and increases the selection pressure for antibiotic resistant strains of the bacteria [12]. In recent years, molecular diagnostic techniques have been developed to increase the efficiency of diagnosing* P. aeruginosa* infection. A majority of these new techniques use polymerase chain reaction (PCR) to identify genes in* P. aeruginosa *[13–16]. Although PCR based diagnostic methods are proven to be sensitive, clinical samples presented may have DNA polymerase inhibitor and other contaminants that increase chances of false positive, which means that a greater amount of time is required to purify samples [17]. Another major limitation of PCR is that it cannot detect and monitor levels of virulence factors, such as membrane antigens and toxins [17]. For example, gene code for Exotoxin A production is not expressed constitutively, due to different environmental factors [18, 19]. Previous studies have demonstrated the clinical role of Exotoxin A in the pathogenesis of* P. aeruginosa* infections [20]. Patients with higher amount of antibodies against Exotoxin A were correlated with better prognosis [21, 22]. This suggests that Exotoxin A is a significant virulence factor of the bacteria and is an important* P. aeruginosa* infection biomarker. However, there is currently lack of regulatory approved Exotoxin A detection methods for diagnosis purpose. Therefore, there is an increasing need to develop new methods to rapidly measure Exotoxin A through molecular recognition and detection, therefore facilitating the diagnosis of* P. aeruginosa* infections.

Systematic Evolution of Ligand by Exponential Enrichment (SELEX) was first described by the Gold laboratory in 1990 [27]. It utilizes an* in vitro* selection process that identifies Molecular Recognitions Elements (MREs) that have very high affinity and specificity to their target molecules. The selection process of nucleic acid MREs usually begins with a library of 10^13^ to 10^15^ different single-stranded DNA (ssDNA) or RNA molecules. The library is then subjected to repeating cycles of partitioning and enrichment for molecules that bind to positive target (target of interest) but not to negative targets. Eventually a single MRE is identified with high specificity and affinity to the target of interest that will be useful for its detection.

In this study, a novel variation of SELEX termed Decoy-SELEX has been utilized for the identification of a single-stranded DNA MRE that binds to Exotoxin A with high affinity and specificity. The advantage of this variation is an increased emphasis on selecting against negative targets. The first negative target, bovine serum albumin (BSA), is selected based on the similarity in structure and amino acid sequence to human serum albumin [28], which is an abundant protein in blood samples. The second negative target, Cholera toxin, served as example of common bacteria virulence factor [29]. The selection scheme is also designed to decrease nonspecific binding to streptavidin and biotin, substrates used in target immobilization. Surface plasmon resonance has been used to characterize the affinity and specificity of the selected MRE. In addition, a modified enzyme-linked immunosorbent assay (ELISA) has been developed by using the selected MRE as the toxin capturing element in human serum and demonstrated the potential use in clinical diagnosis [30–32].

## 2. Materials and Methods

### 2.1. Decoy-SELEX Method for Selection of Exotoxin A Specific MREs

**Figure 1:**
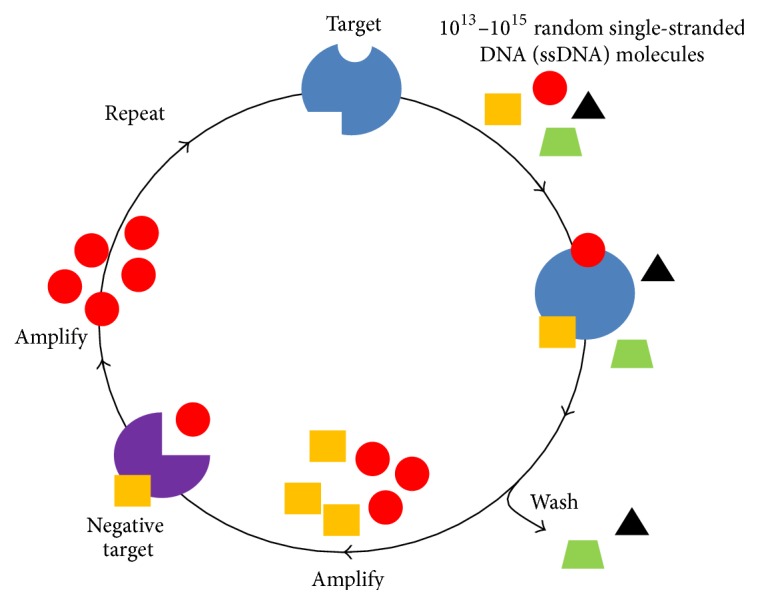
Illustration of the Decoy-SELEX process.* In vitro* selection begins with incubation of target Exotoxin A with a library of 10^15^ ssDNA molecules. Binding molecules are amplified and subjected to incubation with multiple negative targets. Molecules that do not bind to negative targets are amplified and carried on to the next round of selection.

**Figure 2:**
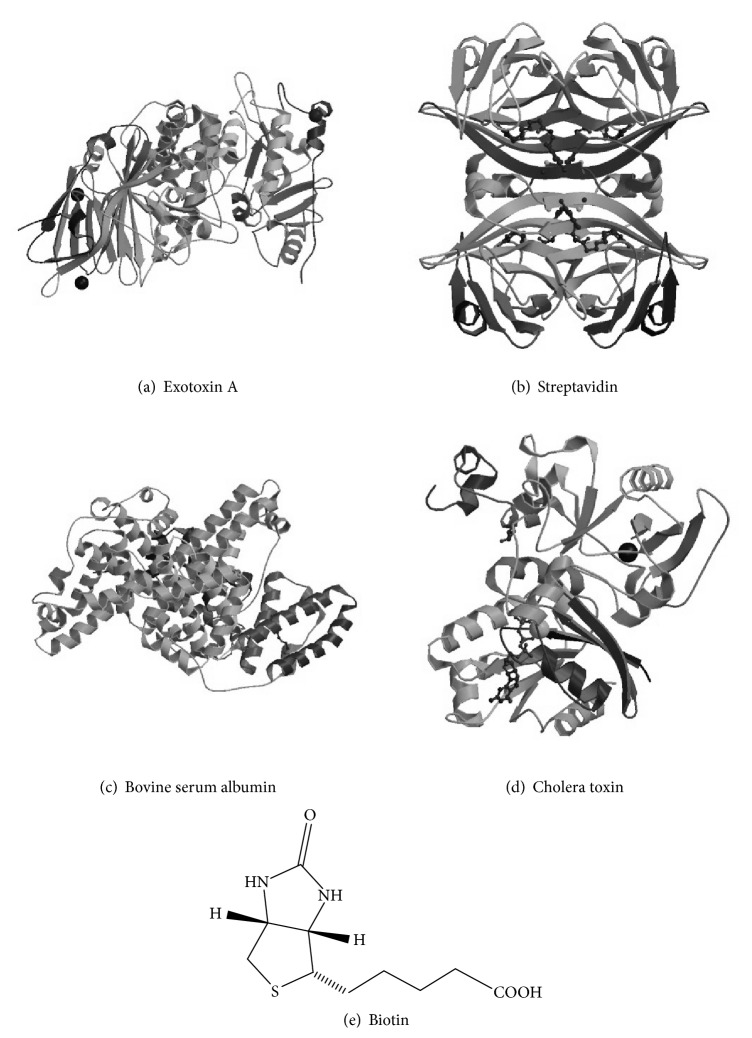
Structures of targets used in the Decoy-SELEX and SPR cross-binding assays. (a) Ribbon structure of the target of interest, Exotoxin A (PDB 1IKQ, 66 kDa) [8]. (b) Ribbon structure of streptavidin (PDB 4GJS, 60 kDa) used in cross bind assays [23]. ((c), (d)) Ribbon structures of bovine serum albumin (PDB 4F5S, 66.5 kDa) and Cholera toxin (PDB 2A5D, 84 kDa) used in negative rounds of selection and crossing binding assays [24, 25]. (e) Chemical structure of biotin used in negative rounds of selection and cross-binding assays.

A single-stranded DNA (ssDNA) library consisting of 10^15^ molecules was used to begin the selection of Exotoxin A specific MREs. This library, named RMW.N34, consisted of two 23-base constant regions for primer annealing flanked by a 34-base random region. It was designed by our laboratory and commercially synthesized (Eurofins MWG Operon; Huntsville, AL, USA). A total of 14 rounds of Decoy-SELEX were utilized to enrich Exotoxin A specific MREs (Table 1) and eliminate MREs that bind to negative targets (Figures 1 and 2).

Exotoxin A in lyophilized powder form (List Biological Laboratories; Campbell, CA, USA) was reconstituted in pure water and then covalently biotinylated via Sulfo-NHS-LC-Biotinylation (Pierce; Rockford, IL, USA) according to manufacturer's protocol. Biotinylated Exotoxin A was washed with ZEBA Spin Desalting Column (Pierce, Rockford, IL, USA) to remove excess unreacted biotin. Subsequently, biotinylated Exotoxin A was bound to streptavidin-coated magnetic beads (New England Biolabs, Ipswich, MA, USA) and washed to generate immobilized target (IT) for selections.

In round 1(+) of selection, 50 *μ*L of IT was incubated with 10^15^ copies of ssDNA from the library in a total of 500 *μ*L of selection buffer composed of 100 mM sodium chloride, 20 mM Tris-HCl, and 2 mM magnesium chloride (1x selection buffer) at room temperature for 46 hours on rotisserie. After the incubation, the selection mixture was subjected to magnetic separation. Unbound ssDNA was removed and ssDNA-bound to IT was washed with 500 *μ*L of selection buffer three times and resuspended in 100 *μ*L of selection buffer. This solution containing IT functioned as template for PCR amplification using the following reaction conditions: enriched ssDNA, 400 nM forward and biotinylated reverse RMW.N34 primers (Eurofins MWG Operon; Huntsville, AL, USA) (forward: 5′-TGTACCGTCTGAGCGATTCGTAC-3′, biotinylated reverse: 5′-Biotin-GCACTCCTTAACACTGACTGGCT-3′), 250 *μ*M deoxynucleotide triphosphates, 1x GoTaq Reaction Buffer (Promega; Madison, WI, USA), 3.5 units of* Taq*, and pure water. Thermal cycling conditions were as follows: first denaturing at 95°C for 5 minutes; cycling at 95°C for 1 minute, 63°C for 45 seconds, and 72°C for 1 minute; and final extension temperature at 72°C for 7 minutes. Large-scale PCR (2 to 4 mL) was performed after each round of positive and negative selection.

After PCR amplification, PCR product containing dsDNA was purified with the GFX PCR purification kit (GE Healthcare, Piscataway, NJ, USA). Eluted dsDNA containing the biotinylated reverse strand was then incubated with streptavidin agarose resin (Pierce; Rockford, IL, USA) for single strand separations [33]. This mixture was transferred into a flow-through column and washed with 5 volumes of 1x phosphate buffer solution. Five volumes of 1 M sodium hydroxide were then added to the column to elute the forward strand of the dsDNA. Subsequently, 0.1 volumes of 3 M sodium acetate at pH 5.2, 2.5 volumes of cold 100% ethanol, and 10 *μ*g/mL of glycogen were added to the eluted ssDNA for ethanol precipitation at −80°C. After the solution was frozen, it was then centrifuged at 13,000 ×g for 1 hour. Precipitated ssDNA was subsequently washed with 70% ethanol and centrifuged at 13,000 ×g for 15 minutes to remove coprecipitated salt. The ssDNA pellet was dried in a vacuum desiccator and resuspended in 50 *μ*L of selection buffer. A NanoDrop spectrometer (ThermoScientific; Wilmington, DE, USA) was used to confirm that the suspension contained at least 10^13^ copies of ssDNA before proceeding to next round of selection.

Round 1(−) was performed by incubating enriched ssDNA from the preceding positive round with immobilization substrate in a total volume of 100 *μ*L selection buffer at room temperature for 18 hours on rotisserie. Immobilization substrate was prepared by incubating Sulfo-NHS-LC-Biotin (Pierce; Rockford, IL, USA) with Tris-HCl and streptavidin-coated magnetic beads (New England Biolabs, Ipswich, MA, USA). After magnetic separation, unbound ssDNA served as the template for PCR amplification as illustrated above. Positive rounds 1 to 7 and negative rounds 1, 2, 4, and 11 were performed as described with decreasing incubation time in positive rounds and increasing incubation time in negative rounds to increase stringency for selection of MREs specific for Exotoxin A.

Starting with round 6(−) of selection, the first negative target, bovine serum albumin (BSA) (Rockland Immunochemical; Gilbertsville, PA, USA), was introduced to the selection. Immobilized negative targets (INT) were prepared identical to IT, substituting Exotoxin A with bovine serum albumin. INT, 50 *μ*L, was incubated with enriched ssDNA from the preceding positive selection round in a total of 500 *μ*L of selection buffer at room temperature for 24 hours. Unbound ssDNA was removed with magnetic separation and served as template for PCR amplification. Round 10(−) was performed in the same way. In round 8(−), the second negative target, Cholera toxin (List Biological Laboratories; Campbell, CA, USA), was introduced. Preparation of Cholera toxin INT was as described above. Selection conditions were similar to round 6(−) with the exception of 18-hour incubation. This was to ensure that the selected MRE was specific to Exotoxin A and not BSA or Cholera toxin.

Starting with round 8(+) of selection, the volume of IT used was decreased in order to increase the stringency of the selection. Rounds 13(+) and 14(+) of IT were subjected to competitive elution with free Exotoxin A solution. IT and the enriched ssDNA were initially incubated for 5 seconds in total of 100 *μ*L of selection buffer. IT with bound ssDNA was washed with 500 *μ*L of selection buffer three times, then 2 *μ*g or 1 *μ*g, respectively, of Exotoxin A in 100 *μ*L of selection buffer was added to the mixture and then incubated for 5 seconds. The supernatant obtained from magnetic separation was used as template for PCR amplification. Round 13(−) was performed in the same way using a free BSA competitive elution; however, ssDNA bound to IT was separated and served as PCR template. This was to ensure ssDNA only binds to free Exotoxin A and not to free negative targets.

### 2.2. Cloning and Sequencing of Exotoxin A Specific MREs

DNA sequencing was performed following rounds 3(+), 6(−), 9(+), 12(+), 13(+), and 14(+) to analyze the ssDNA library for consensus binding sequences. The ssDNA library was amplified with nonbiotinylated primers. It was then ligated into the pCRII vector (Invitrogen, Carlsbad, CA, USA) and transformed into competent* E. coli* bacteria according to manufacturer's protocol. Inserted plasmid was subsequently extracted and purified with the AxyPrep Plasmid Miniprep Kit (Axygen, Union City, CA, USA). The M13R primer, complementary to a region upstream of the PCR insert, was sequenced (Eurofins MWG Operon, Huntsville, AL, USA) along with purified plasmid. A total of 30–50 randomly selected sequences for each respective round were subjected to analysis for consensus sequence families [34, 35].

### 2.3. Exotoxin A MRE Binding Assays with Surface Plasmon Resonance

After analyzing round 14 for its DNA sequences, R14.33 was selected for further characterization. The Mfold DNA web server was used to predict the secondary structure with the following conditions: 25°C, 100 mM Na^+^, and 2 mM Mg^2+^ [26]. Subsequently, R14.33 was synthesized by Eurofins MWG Operon with a 5′ amino-C6 modification for use in surface plasmon resonance (SPR) binding assays.

Glass slides (12 mm × 10 mm) were cleaned by sonication in acetone, isopropyl alcohol, and doubly deionized water (5 min, each) and then blown dry with nitrogen. Gold was evaporated onto the slides using Temescal BJD-2000 system (Edwards Vacuum; Phoenix, AZ, USA) with an Inficon XTC/2 deposition controller (Inficon; East Syracuse, NY, USA) (chamber pressures ≤ 1.0 × 10^−6^ Torr). Samples were rotated (3 rpm) and monitored during deposition for metal thickness (6 MHz quartz piezoelectric crystals, gold-coated) (Kurt J. Lesker Co., Clairton, PA, USA). Rates of 0.3−0.5 Å/s were maintained during the deposition of a titanium adhesion layer (2 nm) and a gold layer (50 nm). After that, samples were cooled to room temperature before being removed from the chamber.

The gold slide was then cleaned in 100% ethanol under sonication for 5 minutes and then placed in a solution containing 10 mM 11-mercaptoundecanoic acid (11-MUA) (Sigma; St. Louis, MO, USA) and 10 mM triethylene glycol mono-11-mercaptoundecylether (PEG3) (Sigma; St. Louis, MO, USA) in a 1 to 5 ratio for 24 hours under argon. After the formation of the self-assembled monolayer (SAM), the gold slide was rinsed with 100% ethanol, pure water, and blown dry with a slow stream of nitrogen. The prepared gold slide was inserted into the carrying cartridge and docked into a Biacore X100 (GE Healthcare; Piscataway, NJ, USA). The running buffer for immobilization was composed of 100 mM sodium chloride, 20 mM potassium phosphate, and 2 mM magnesium chloride, pH 7.56 (1x immobilization buffer). Next, 100 mM N-hydroxysulfonyl succinimide (sulfo-NHS) (Pierce; Rockford, IL, USA) and 400 mM 1-ethyl-3-(3-dimethylaminopropyl) (EDC) (Pierce; Rockford, IL, USA) were mixed (1 : 1) and injected into flow cell 1 and 2 at a flow rate of 5 *μ*L/min for ten minutes for the activation of the carboxyl group of 11-MUA. Then, 300 *μ*L of 1 *μ*M 5′ amino modified ssDNA in immobilization buffer (after denaturing at 95°C for 5 min and cooling to room temperature) was injected into flow cell 2 at a rate of 5 *μ*L/min. At the end of the DNA injection, unreacted carboxyl groups were inactivated by injection of selection buffer twice for a total of twenty minutes, followed by a regeneration cycle with 45 mM glycine and 100 mM sodium hydroxide in 5% ethanol (regeneration buffer) for 30 seconds [36, 37].

After immobilization, selection buffer was then used as the running buffer for binding assays. The binding affinity of R14.33 was determined by injecting concentrations of 0, 0.5, 1, 1.2, 1.4, and 2 *μ*M of Exotoxin A in flow cells 1 and 2 at a flow rate of 5 *μ*L/min at room temperature. Each cycle comprised a 180-second wait period, 180-second contact period, 180-second wait period, and 30-second regeneration period using regeneration buffer. Assays were performed in duplicate [36, 37]. Kinetic data was analyzed using the Scrubber-2 software (BioLogic Software; Campbell, Australia) to determine the equilibrium dissociation constant (*K*_*d*_), assuming a one-to-one kinetics model.

To determine the cross-binding activity of R14.33 to negative targets, blank selection buffer and 5 *μ*M each of Exotoxin A, BSA, biotin (Sigma; St. Louis, MO, USA), Cholera toxin, and streptavidin (Amresco; Solon, OH, USA) were injected into both flow cells with the same conditions as described above. Each molecule was tested in triplicate. All data was averaged and standard deviations were calculated as previously described [36]. One-way ANOVA and Bonferroni post hoc test were performed to determine statistical differences in the means for analytes.

### 2.4. Exotoxin A MRE Modified ELISA Assays

A sandwich ELISA assay modified with ssDNA MRE was developed. R14.33 was commercially synthesized with 5′ biotinylation for the use as the antigen capturing element (Eurofins MWG Operon; Huntsville, AL, USA). Streptavidin-coated 96-well plate (Pierce; Rockford, IL, USA) was washed three times for 5 minutes, with 200 *μ*L of wash buffer (1x selection buffer, 0.1% BSA, 0.05% Tween-20 detergent). Subsequently, 100 *μ*L of 40 nM 5′ biotinylated ssDNA in selection buffer (after denaturing at 95°C for 5 min and cooling to room temperature) was added to sample wells and incubated for 2 hours with shaking at room temperature. Each well was washed three times with wash buffer to remove nonimmobilized ssDNA. A negative control for each replicate consisted of a blank well without immobilized ssDNA. Then, 100 *μ*L of each 1x phosphate buffer solution, selection buffer, 100 nM of Exotoxin A in selection buffer, human serum (Sigma; St. Louis, MO, USA), or 100 nM of Exotoxin A in human serum was added to individual sample wells. The plate was incubated for 1 hour with shaking at room temperature.

Following sample incubation, each well was washed three times with wash buffer to remove unbound Exotoxin A. Next, 100 *μ*L of 1 : 100 dilution of primary goat anti-Exotoxin A antibody (List Biological Laboratories; Campbell, CA, USA) in wash buffer was added to each well and followed by 30 minutes of incubation with shaking at room temperature. Following primary antibody incubation, each well was washed three times. Subsequently, 100 *μ*L of 1 : 500 dilution of secondary rabbit anti-goat antibody conjugated to horseradish peroxidase (Pierce; Rockford, IL, USA) in wash buffer was added to each well and incubated for 30 minutes at room temperature with shaking. Lastly, each well was washed five times to remove nonspecifically bound antibodies. Controls without antibodies and with only primary antibodies added were also performed. Assays were performed in quadruplicate.

ABTS substrate (Pierce; Rockford, IL, USA) was added to each well according to the manufacturer's instruction. After ABTS was added, absorbance was measured in a Synergy 2 microplate reader with OD reading at 410 nm and 650 nm using Gen5 1.06 software (Biotek US; Winooski, VT, USA) in two-minute increments. All data was averaged and standard deviations were calculated. For each Exotoxin A containing sample, a two-tailed Student's *t*-test was performed to determine statistical differences, respectively, to selection buffer or human serum blank controls at *P* < 0.05.

## 3. Results and Discussions

### 3.1. Selection of Exotoxin A Specific MREs

Fourteen rounds of Decoy-SELEX were performed to identify ssDNA MREs specific to Exotoxin A (Table 1). The selection scheme was aimed to direct the ssDNA MREs to bind to free Exotoxin A in solution and reduce enrichment of nonspecific binding to immobilization substrates, BSA, and Cholera toxin. Initially, 30–50 randomly selected sequences were analyzed for the presence of consensus sequence families after rounds 3, 6, 9, and 12. Toward the end of the selection, in order to monitor the convergence of families more frequently, 30–50 random sequences from both rounds 13 and 14 were analyzed as previously described [34, 35, 38].

**Figure 3:**
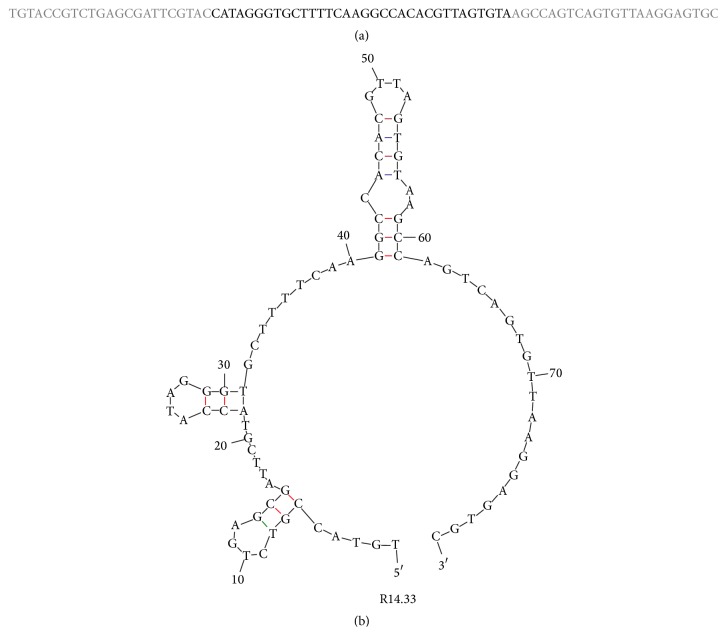
Secondary structure and sequence of R14.33 ssDNA MRE. (a) ssDNA sequence of Exotoxin A MRE R14.33. Gray letters indicate constant regions of the MRE. (b) Mfold prediction of R14.33 secondary structure [26].

In the round 14 of ssDNA library, there was a noticeable and significant presence of sequence families in one sequence, R14.33 (Table 2). This sequence appeared in about 40% of the sequence families. R14.33 had one possible predicted structure, with a relatively low Gibbs free energy value of −9.93 kcal/mol according to the Mfold prediction. This indicated a relatively stable secondary structure at the given conditions (Figure 3). The variable region of R14.33 also participated in the formation of two stem-loop structures [39]. Therefore, R14.33 was chosen for further characterization.

### 3.2. Affinity and Specificity of Exotoxin A Specific MRE

**Figure 4:**
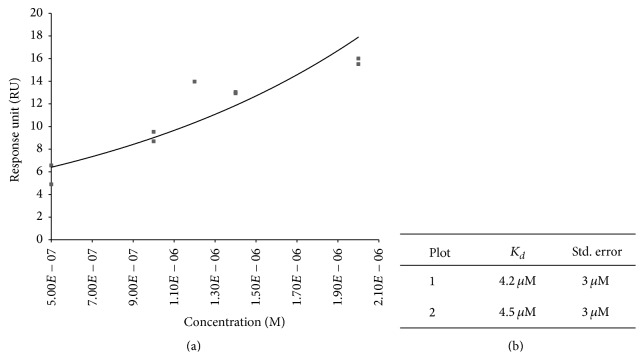
SPR binding kinetics assays of R14.33. Data represent *K*_*d*_ of R14.33 from two binding assays evaluated via Scrubber 2 software (Software; Campbell, Australia). (a) Representative binding response curve of R14.33. (b) Equilibrium dissociation constants and standard error of two binding assays.

Affinity of the selected MRE was determined by SPR binding assays. Assays were performed with Exotoxin A concentration from high nM to low *μ*M range with at least 2 duplicate concentrations. The equilibrium dissociation constant (*K*_*d*_) was between 4.2 and 4.5 *μ*M (Figure 4). In recent years, there have been a number of MREs selected against protein targets which utilized SPR for characterization of binding affinity. Reported equilibrium dissociation constants in these studies range from low-nanomolar to high-nanomolar [40–44]. It is to be noted that these studies relied on different immobilization methods compared to what was used here, including streptavidin-biotin linkage and thiolated DNA attachment [41–44]. Also, several studies utilized a sandwich detection method to amplify signals, thus enhancing the limit of detection [42, 45, 46].

**Figure 5:**
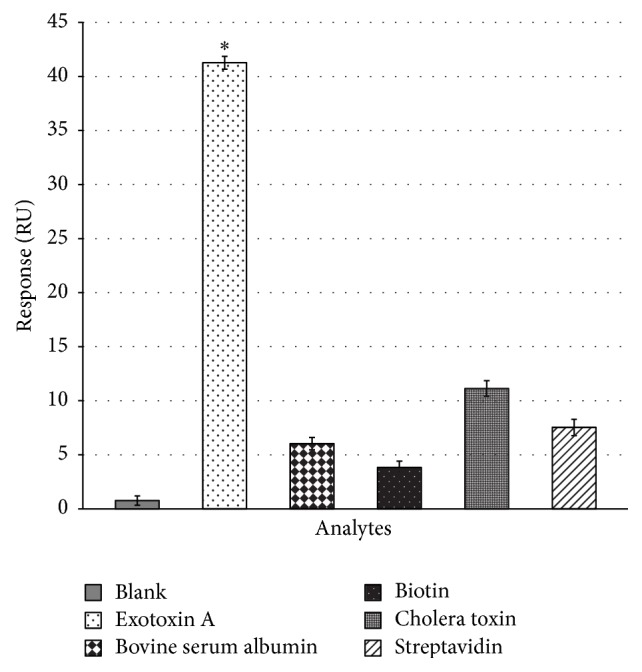
SPR cross-binding assays of R14.33. Data represent specificity of R14.33 Exotoxin A MRE. Error bars represent standard deviations of three runs. Statistical significance levels of *P* < 0.001 are designated by “*∗*”. The observed significance levels are adjusted by Bonferroni post hoc procedure. Exotoxin A has a significantly higher response when compared to blank control and all other analytes, indicating low cross-binding activities of R14.33. Blank represents 1x selection buffer. Concentrations of all analytes are at 5 *μ*M.

SPR cross-binding assays were performed to test the specificity of the selected Exotoxin A MRE. Concentrations of all cross-binding analytes were higher than those used in affinity assays to ensure that R14.33 has no nonspecific binding to negative targets. The method of presentation of the current data as relative response unit of R14.33 to all analytes has been previously described [36, 46]. Binding responses showed a much higher affinity of R14.33 to Exotoxin A in solution than to all negative targets (one-way ANOVA: *F*_2,12_ = 573.4, *P* < 0.001) (Figure 5), and to streptavidin, a significant component of the immobilization substrate. This is noteworthy as streptavidin was present in all of the selection rounds. It is clear that competitive elutions performed in the last two positive rounds gave the ssDNA library selectivity for free Exotoxin A in solution over immobilized target and other negative targets, thus validating the Decoy-SELEX method.

The determined equilibrium binding constant of the selected Exotoxin A MRE is higher than other studies utilizing SPR binding assays. This difference is likely due to different methods of immobilizing the ssDNA MRE as noted above and thus leads to a lower level of immobilization and detection responses. The current study utilizes direct covalent conjugation of 5′ amino modified ssDNA to the SAM on gold surface [34, 35, 37]. However, covalently attached DNA provides a more stable immobilization as compared to streptavidin/biotin and thiolated DNA attachment under a wide range of storage conditions. This is a potential advantage in the real application of a ssDNA MREs based biosensor, as the longevity of the biological probe is a huge determining factor of its application value [47]. It is also important to note that previous study demonstrated a 100-fold higher *K*_*d*_ between SPR binding measurements and binding assays relying on free ssDNA in solution [48]. This is likely due to the difference in the availability of binding pockets on MREs that are immobilized on solid platform as compared to being in solution. The SPR setup in this study is also very similar to the potential design of a final sensor. Therefore, this is a very realistic assay and is translational as a sensor.

### 3.3. Diagnostic Application of Exotoxin A Specific MRE

**Figure 6:**
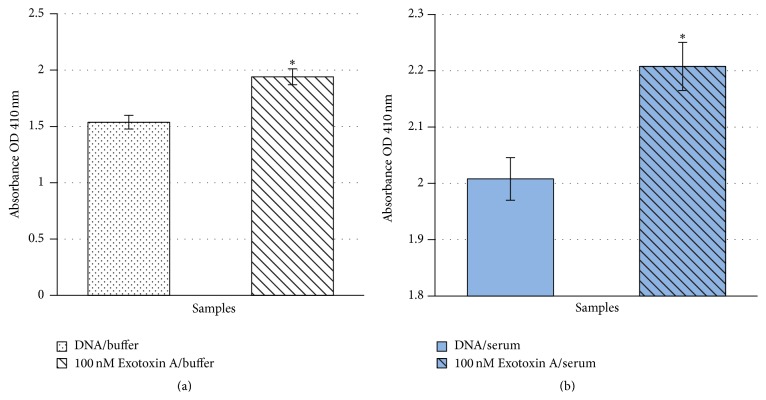
Modified ELISA assays of Exotoxin A. Data represent one modified sandwich ELISA with absorbance measured at OD 410 nm. Absorbance levels presented are subtracted from background levels of blank well without immobilized DNA. Error bars represent standard deviations of 4 sample replicates in one independent assay. (a) Statistical significance levels with respect to buffer background of *P* < 0.001 are designated by “*∗*”. (b) Statistical significant levels with respect to human serum background of *P* < 0.001 are designated by “*∗*”. Buffer: 1x selection buffer; serum: human serum.

The Exotoxin A specific MRE demonstrated high specificity and minimal cross-binding activity to BSA. It is reasonable to believe that this low binding property may be extrapolated to human serum albumin [41]. This allowed the investigation of using the selected MRE as a potential diagnostic tool. A sandwich ELISA assay modified with the ssDNA MRE as the toxin capturing element was developed. Reproducible and statistically significant detection of Exotoxin A at 100 nM in spiked human serum samples was achieved compared to negative controls in six independent assays (*P* < 0.05 to *P* < 0.001) (Figure 6). It has been reported that ssDNA MRE generally has a half-life of 1 hour in human serum due to the presence of exonuclease [49]. Therefore, toxin incubation time that ranged from five minutes to one hour was tested during assay development. While a portion of the immobilized MRE is likely to be degraded in serum condition, the one-hour toxin incubation period yielded the most consistent result and therefore it was utilized in all experimental assays.

Previous study has attempted to incorporate ssDNA MRE into a system for target detection in buffer diluted human serum [50]. Similar ssDNA MRE modified ELISA assay for the detection of bacteria toxins has been previously described [51, 52]. However, clinically relevant samples were not tested in both studies. It is known that the binding activities of nucleic acid MREs are highly dependent on their three-dimensional structures and are influenced by factors such as temperature, pH, and ionic strength of the binding environment [39]. This leads to challenges in applying nucleic acid MREs in targets' native complex environments, such as human serum [53]. This study demonstrated an improvement to these previous reported studies by showing the robustness of the selected Exotoxin A specific MRE in undiluted serum without any base modifications, and it was able to retain a level of binding activity in an environment that was very much different compared to the selection condition.

Currently, there are limited studies in quantifying the amount of Exotoxin A* in vivo*. Previous studies showed large differences in the levels of Exotoxin A detected in murine serum (averaged 116.0 ng/mL) and in culture media (averaged 1.4 *μ*g/mL) [54, 55]. One study showed significant differences in Exotoxin A detected in different patient sputum samples (0.3 ng/mL to 126 ng/mL), and as high as 29.3 *μ*g/mL of Exotoxin A was detected in the culture supernatant of sputum isolated* P. aeruginosa* [56]. It has also been reported that blood isolated* P. aeruginosa* produced the highest amount of Exotoxin A in culture conditions (approximately 0.3 *μ*g/mL) [57]. Overall, these results suggest that* in vivo* levels of Exotoxin A vary significantly and are not well quantified in human patients.

Historically, an ADP-ribosylation assay has been utilized to quantify the amount of Exotoxin A in research studies [20, 57–60]. This assay requires the use of radioactive NAD and extensive experimental preparations, and therefore it may not be practical for diagnostic use. Although a traditional antibody-based sandwich ELISA assay (MyBioSource; San Diego, CA, USA) is commercially available with a reported detection range between 0.156 ng/mL and 10 ng/mL, it was designed for research purposes only. Also, the ranges of Exotoxin A levels* in vivo* are likely to be wide. The relatively narrow detection range of the commercial ELISA kit may limit its usage in clinical samples. It is to be noted that, in our ssDNA MRE modified ELISA assays, 100 nM or 6.6 *μ*g/mL of Exotoxin A in human serum was detected. While this is highly reproducible, the differences compared to negative controls are small enough to be near the assay's detection limit. Based upon the available clinical data on Exotoxin A level in patients, it is difficult to completely rule out the clinical usage of the current ssDNA MRE modified ELISA assay. In order to transition the current assay into a final product for clinical use, it will likely require modification, optimization, and possibly an industrial partnership for development. Nevertheless, ssDNA MRE modified assay does offer several advantages when compared to ELISA assays that are based solely on antibodies, such as thermostability, and regeneration of assay by a basic buffer wash [61].

Overall, this study has identified a ssDNA MRE with high affinity and specificity for Exotoxin A of* P. aeruginosa*. To the best of our knowledge, this is the first ssDNA MRE targeting Exotoxin A. The successful use of SPR for MRE characterizations showed the potential of it being incorporated into a SPR-based biosensor for real time, label-free detection of Exotoxin A in biological matrices [41, 42]. In addition, the ssDNA MRE modified ELISA assay offers a potential new way to facilitate the diagnosis of* P. aeruginosa* infection by rapidly identifying the presence of one of the most significant virulence factors. The ELISA requires minimal sample manipulations and approximately two hours from toxin incubation to detection. This method may also supplement direct diagnosis methods based on detecting the presence of bacterial cells through culturing and PCR.

## 4. Conclusions 

This study utilized a novel variation of the SELEX process, Decoy-SELEX, to obtain a ssDNA Molecular Recognition Element specific for Exotoxin A, a virulence factor of* Pseudomonas aeruginosa*. The MRE is characterized to have high affinity and specificity to its target, thus validating the Decoy-SELEX methodology. It also showed sensitive detection of Exotoxin A at nanomolar concentrations in human serum through a modified sandwich ELISA assay and demonstrated the proof-of-principle diagnostic application of ssDNA MREs.

## Figures and Tables

**Table 1:** Decoy-SELEX scheme for Exotoxin A MRE selection.

Round	Positive selection (+)	Negative selection (−)
1	Immobilized target (IT) 46 hrs, 50 *µ*L	Immobilization substrate (IS) 18 hrs, 50 *µ*L
2	IT 24 hrs, 50 *µ*L	IS 22 hrs, 50 *µ*L
3	IT 18 hrs, 50 *µ*L	—
4	IT 12 hrs, 50 *µ*L	IS 20 hrs, 50 *µ*L
5	IT 8 hrs, 50 *µ*L	—
6	IT 5.5 hrs, 50 *µ*L	BSA immobilized negative target (INT) 24 hrs, 50 *µ*L
7	IT 1 hrs, 50 *µ*L	—
8	IT 1 hrs, 25 *µ*L	Cholera toxin INT 18 hrs, 50 *µ*L
9	IT 1 hrs, 5 *µ*L	—
10	IT 5 min, 5 *µ*L	BSA INT 24 hrs, 50 *µ*L
11	IT 5 sec, 5 *µ*L	IS 21 hrs, 10 *µ*L
12	IT 5 sec, 5 *µ*L	—
13	IT 5 sec, 5 *µ*L; competitive elution with 2 *µ*g free Exotoxin A, 5 sec	IT 5 sec, 5 *µ*L; competitive elution with 1 mg/mL free BSA, 5 min
14	IT 5 sec, competitive elution with 1 *µ*g free Exotoxin A, 5 sec	—

*In vitro *selection performed for identifying Exotoxin A specific MRE. Immobilized target (IT) is Exotoxin A bound to magnetic beads. Immobilization substrate (IS) is streptavidin-coated magnetic beads blocked with biotin regent. BSA is the abbreviation for bovine serum albumin. Times listed are incubation times in hours (hrs), minutes (min), or seconds (sec).

**Table 2:** Sequence families after round 14 Decoy-SELEX.

R14.02	TGTACCGTCTGAGCGATTCGTACTAC$\underline{\underline{GCCA}CACGT}$GGTGAGGGATTCGATCGCTTGAAGCCAGTCAGTGTTAAGGAGTGC
R14.20	TGTACCGTCTGAGCGATTCGTACTGCTATTCATCACCACTCTAGA$\underline{\underline{GCCA}}$CTTTTAAAAGCCAGTCAGTGTTAAGGAGTGC
R14.22	TGTACCGTCTGAGCGATTCGTACTGGGCGGCGA$\underline{\underline{GCCA}}$CCCGGCAATTTAGTACAGGCAGCCAGTCAGTGTTAAGGAGTGC
R14.33	TGTACCGTCTGAGCGATTCGTACCATAGGGTGCTTTTCAAG$\underline{\underline{GCCA}CACGT}$TAGTGTAAGCCAGTCAGTGTTAAGGAGTGC
R14.36	TGTACCGTCTGAGCGATTCGTACAAAGCATCCAGCCGGTATGT$\underline{\underline{GCCA}}$GAGTCTCTGAAGCCAGTCAGTGTTAAGGAGTGC
R14.38	TGTACCGTCTGAGCGATTCGTACAAATGTGAGTG$\underline{\underline{GCCA}}$GGCATCAGGTACGTCGGTAAGCCAGTCAGTGTTAAGGAGTGC

R14.03	TGTACCGTCTGAGCGATTCGTACGGATAGGTGCCTCTGC$\underline{\underline{TTCA}}$TCATGTTGAACTTAAGCCAGTCAGTGTTAAGGAGTGC
R14.13	TGTACCGTCTGAGCGATTCGTACAGT$\underline{\underline{TTCA}}$CCAGTCGCCTGTTAGCCGTGATATACGAGCCAGTCAGTGTTAAGGAGTGC
R14.20	TGTACCGTCTGAGCGATTCGTACTGCTA$\underline{\underline{TTCA}}$TCACCACTCTAGAGCCACTTTTAAAAGCCAGTCAGTGTTAAGGAGTGC
R14.27	TGTACCGTCTGAGCGATTCGTACTGTCAATATTACGTTGCTCTTAGG$\underline{\underline{TTCA}}$CCATCTAGCCAGTCAGTGTTAAGGAGTGC
R14.28	TGTACCGTCTGAGCGATTCGTACTTGTGA$\underline{\underline{TTCA}}$AATAGGCGTGTTG$\underline{GTGT}$GAGACCTAGCCAGTCAGTGTTAAGGAGTGC
R14.33	TGTACCGTCTGAGCGATTCGTACCATAGGGTGCTT$\underline{\underline{TTCA}}$AGGCCACACGTTA$\underline{GTGT}$AAGCCAGTCAGTGTTAAGGAGTGC

R14.03	TGTACCGTCTGAGCGATTCGTACGG$\underline{\underline{ATAGG}TGC}$CTCTGCTTCATCATGTTGAACTTAAGCCAGTCAGTGTTAAGGAGTGC
R14.23	TGTACCGTCTGAGCGATTCGTACCGTGGATCATGCTTCGCGTCGGTTT$\underline{\underline{ATAGG}}$TTCCAGCCAGTCAGTGTTAAGGAGTGC
R14.33	TGTACCGTCTGAGCGATTCGTACC$\underline{\underline{ATAGG}GTGC}$TTTTCAAGGCCACACGTTAGTGTAAGCCAGTCAGTGTTAAGGAGTGC
R14.39	TGTACCGTCTGAGCGATTCGTACAGAAGAGCATCGGTAACTTCC$\underline{\underline{ATAGG}}$AGATGGGGAGCCAGTCAGTGTTAAGGAGTGC

R14.02	TGTACCGTCTGAGCGATTCGTACTACGCCACACGTGGTG$\underline{\underline{AGGG}}$ATTCGATCGCTTGAAGCCAGTCAGTGTTAAGGAGTGC
R14.06	TGTACCGTCTGAGCGATTCGTACCATCCG$\underline{\underline{AGGG}}$TATTGTATGCGTATATCCTAGTCGAGCCAGTCAGTGTTAAGGAGTGC
R14.10	TGTACCGTCTGAGCGATTCGTACCAAGTTCCTCATGG$\underline{\underline{AGGG}TGCT}$CAGAGCTTAGACAGCCAGTCAGTGTTAAGGAGTAA
R14.33	TGTACCGTCTGAGCGATTCGTACCAT$\underline{\underline{AGGG}TGCT}$TTTCAAGGCCACACGTTAGTGTAAGCCAGTCAGTGTTAAGGAGTGC
R14.34	TGTACCGTCTGAGCGATTCGTAC$\underline{\underline{AGGG}}$GGATTCCT$\underline{\underline{AGGG}}$CCCGGCCCAACGCTGTTTAGCCAGTCAGTGTTAAGGAGTGC
R14.42	TGTACCGTCTGAGCGATTCGTACCAAGACCCTTGAATCACGGGT$\underline{\underline{AGGG}}$TCTCGTAACAGCCAGTCAGTGTTAAGGAGTGC

Representative sequence families following round 14 of Decoy-SELEX. Families are grouped by boxes with common sequences double-underlined and subfamilies underlined.
